# Value of the First Post-Transplant Biopsy for Predicting Long-Term Cardiac Allograft Vasculopathy (CAV) and Graft Failure in Heart Transplant Patients

**DOI:** 10.1371/journal.pone.0036100

**Published:** 2012-04-25

**Authors:** Carlos A. Labarrere, John R. Woods, James W. Hardin, Gonzalo L. Campana, Miguel A. Ortiz, Beate R. Jaeger, Lee Ann Baldridge, Douglas E. Pitts, Philip C. Kirlin

**Affiliations:** 1 Experimental Pathology, Methodist Research Institute, Indiana University Health Methodist Hospital, Indianapolis, Indiana, United States of America; 2 Outcomes Research, Methodist Research Institute, Indiana University Health Methodist Hospital, Indianapolis, Indiana, United States of America; 3 Epidemiology and Biostatistics, University of South Carolina, Columbia, South Carolina, United States of America; 4 Dr. Stein und Kollegen, Mönchengladbach, Germany; 5 Department of Pathology and Laboratory Medicine, Indiana University School of Medicine, Indianapolis, Indiana, United States of America; 6 St Vincent Medical Group, St Vincent Hospital, Indianapolis, Indiana, United States of America; 7 Transplant Center, Indiana University Health Methodist Hospital, Indianapolis, Indiana, United States of America; Université Paris Descartes, France

## Abstract

**Background:**

Cardiac allograft vasculopathy (CAV) is the principal cause of long-term graft failure following heart transplantation. Early identification of patients at risk of CAV is essential to target invasive follow-up procedures more effectively and to establish appropriate therapies. We evaluated the prognostic value of the first heart biopsy (median: 9 days post-transplant) versus all biopsies obtained within the first three months for the prediction of CAV and graft failure due to CAV.

**Methods and Findings:**

In a prospective cohort study, we developed multivariate regression models evaluating markers of atherothrombosis (fibrin, antithrombin and tissue plasminogen activator [tPA]) and endothelial activation (intercellular adhesion molecule-1) in serial biopsies obtained during the first three months post-transplantation from 172 patients (median follow-up = 6.3 years; min = 0.37 years, max = 16.3 years). Presence of fibrin was the dominant predictor in first-biopsy models (Odds Ratio [OR] for one- and 10-year graft failure due to CAV = 38.70, p = 0.002, 95% CI = 4.00–374.77; and 3.99, p = 0.005, 95% CI = 1.53–10.40) and loss of tPA was predominant in three-month models (OR for one- and 10-year graft failure due to CAV = 1.81, p = 0.025, 95% CI = 1.08–3.03; and 1.31, p = 0.001, 95% CI = 1.12–1.55). First-biopsy and three-month models had similar predictive and discriminative accuracy and were comparable in their capacities to correctly classify patient outcomes, with the exception of 10-year graft failure due to CAV in which the three-month model was more predictive. Both models had particularly high negative predictive values (e.g., First-biopsy vs. three-month models: 99% vs. 100% at 1-year and 96% vs. 95% at 10-years).

**Conclusions:**

Patients with absence of fibrin in the first biopsy and persistence of normal tPA in subsequent biopsies rarely develop CAV or graft failure during the next 10 years and potentially could be monitored less invasively. Presence of early risk markers in the transplanted heart may be secondary to ischemia/reperfusion injury, a potentially modifiable factor.

## Introduction

Modern immunosuppressive regimens have reduced the incidence of acute rejection and extended early survival following heart transplantation but have done little to reduce the incidence of cardiac allograft vasculopathy (CAV), the principal long-term cause of graft failure. CAV, an aggressive form of atherosclerosis that develops within months to a few years after transplantation, accounts for 30% of all deaths [1]. Because heart transplant patients lack premonitory symptoms, CAV first presents clinically as a silent myocardial infarction, severe arrhythmia, or sudden death. Thus, research has focused on identifying early predictors of CAV onset and progression.

The Invasive Monitoring Attenuation through Gene Expression (IMAGE) trial recently showed that patients at low risk of rejection can be monitored safely with noninvasive gene-expression profiling [2]. It might be possible to devise a similar noninvasive strategy to monitor CAV, provided that low-risk patients could be reliably identified. We recently showed that absence of atherothrombotic risk markers in the first three months post-transplantation identifies patients that rarely develop CAV, suggesting that they might be candidates for less invasive monitoring [3]. This finding led us to study the predictive value of the *first* biopsy, obtained 7–12 days post-transplant. Thus, the aim of this study was to determine whether very early data from a single biopsy are sufficient to identify low-risk patients. Our analysis showed that patients with absence of fibrin in the first biopsy rarely develop CAV or graft failure during the next 10 years. Furthermore, the high negative predictive value of the first-biopsy was comparable to that of multiple biopsies obtained over three-months, implying that patients with negative findings in the first biopsy potentially could be monitored less invasively, thereby, avoiding the risk and expense of multiple heart biopsy procedures.

## Materials and Methods

### Patients

Consecutive adult heart-transplant recipients transplanted from August 1989 to August 2004 and followed prospectively until September 2010, were candidates for study. Patients (n = 172) were included if they survived at least three months post-transplantation, had serial endomyocardial biopsies performed in the first three months, and had their coronary arteries examined angiographically and/or histopathologically for CAV at annual follow-ups. Of 241 candidates, 29 patients were excluded because they had missing three-month biopsy data, either because they died prior to three months (n = 14) or because they were transplanted at another institution (n = 15); 38 survived three-months but were excluded because they had incomplete biopsy data; and two survived but were excluded because of missing follow-up coronary evaluations. The study protocol was approved by the Indiana University local Institutional Review Board and all subjects signed a consent form.

### Clinical management

All patients received triple-drug immunosuppression [4]. Rejection grades 2R-3R [5] were treated with steroids plus rabbit antithymocyte globulin or OKT3 monoclonal antibody. Higher dose immunosuppressants and clinical treatment strategies were used at the physician's discretion without knowledge of immunohistochemical data regarding markers of atherothrombosis and endothelial activation.

Baseline (time-zero) endomyocardial biopsies were performed on all of the 172 donor hearts at the time of transplantation but before reperfusion. Additional biopsies were performed serially during the first three months after transplantation, with the first post-transplant biopsy obtained within a median 9 days of transplantation.

Cytomegalovirus disease was defined during follow-up by clinical symptoms and by cytopathologic-tissue culture evidence of invasion. Cytomegalovirus prophylaxis with gancyclovir was used in seronegative recipients of seropositive donors.

### Outcome Criteria

CAV was evaluated in side-by-side comparisons of identical projections of serial angiograms performed annually (Mean: 5.25±1.0/patient) and diagnosed by evidence of coronary artery narrowing or luminal irregularities either in left main or any primary or branch vessels. CAV was considered severe if left main stenosis was >70%, if two or more primary vessels had stenoses >70%, or if branch stenoses were >70% in all three systems [6]. Presence and severity of CAV were determined by consensus of two experienced angiographers unaware of immunohistochemical biopsy results. In recipients who died before their first annual angiogram, coronary arteries were examined histopathologically and severe CAV was identified using similar criteria to those described for angiographic evaluation. Graft failure due to CAV was defined as: (a) death associated with CAV-related cardiac allograft dysfunction, or (b) need for a second transplant due to severe CAV.

### Immunohistochemistry studies

Endomyocardial biopsies were tested immunohistochemically for fibrin (NYBT2G1, Accurate, Westbury, NY; a-Fib Beta, American Diagnostica, Stamford, CT); tissue plasminogen activator (tPA, ESP-1, American Diagnostica); antithrombin (A0296, DAKO, Carpinteria, CA) and intercellular adhesion molecule-1 (ICAM-1, LB-2, Santa Cruz Biotechnology, Santa Cruz, CA). Affinity-purified, fluorochrome-labeled or peroxidase-labeled polymer conjugated anti-mouse or fluorochrome-labeled anti-rabbit F(ab')_2_ fragments served as secondary antibodies (Molecular Probes, Eugene, OR and Protos ImmunoResearch, Burlingame, CA). Arteries were identified with fluorescein-labeled mouse monoclonal antibody to human smooth muscle α-actin (1A4, Sigma-Aldrich, St Louis, MO).

For immunofluorescence studies, tissue samples were embedded in optimum cutting temperature compound (Miles, Elkhart, IN), snap-frozen in liquid nitrogen and stored at −75°C. Cryostat sections (4 µm) were air-dried overnight without fixation, and immunostained. Rhodamine-conjugated anti-mouse and Alexa Fluor 488 anti-rabbit or anti-mouse were used as secondary antibodies.

For immunoperoxidase studies, slides from paraffin blocks were antigen-retrieved using DAKO Target Retrieval solution (pH 6.0) to expose antigens masked by formalin. Endogenous biotin was blocked with avidin/biotin blocking system (DAKO) and endogenous peroxidase with 3% hydrogen peroxide. Fibrin antibody was applied for 60 minutes at room temperature. Slides were developed using DAKO's EnVision+ Dual Link, HRP kit for mouse primary antibodies in a DAKO Autostainer. Immunohistochemical data were evaluated by two investigators unaware of clinical outcome.

### Coding of biopsy markers

**Figure 1:**
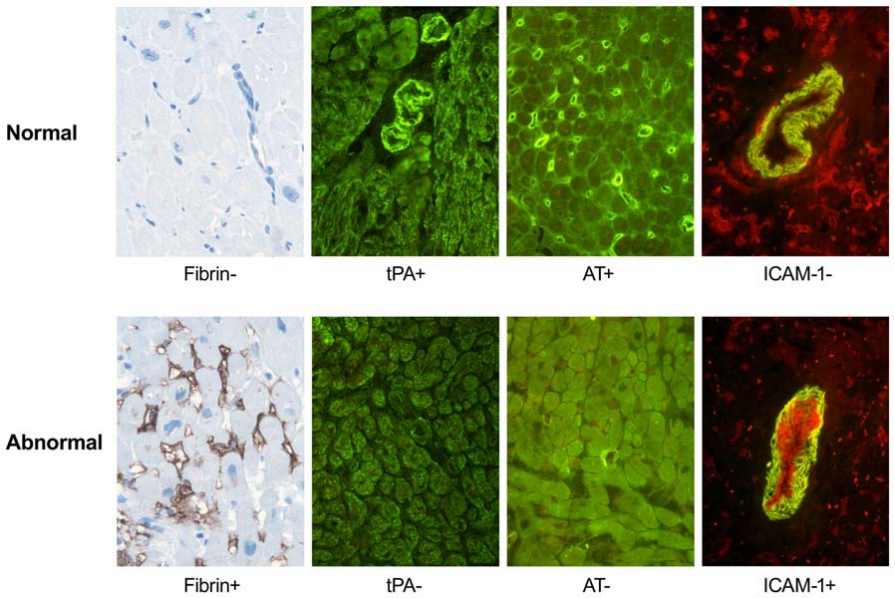
Immunohistochemical characteristics of a thrombotic/activated microvasculature. Normal hearts (top row) have absence of fibrin (Fib−), presence of microvascular antithrombin (AT+) and tissue plasminogen activator (tPA+) and absence of arterial endothelial ICAM-1 (ICAM-1−). Abnormal thrombotic and activated hearts (bottom row) are characterized by presence of fibrin (Fib+), loss of microvascular antithrombin (AT−) and tissue plasminogen activator (tPA−) and expression of arterial endothelial ICAM-1 (ICAM-1+). Original magnification ×640.

Immunohistochemical data were scored as described by Labarrere et al [3]. As illustrated in Figure 1 (top row), normal hearts have absence of fibrin (Fib−), presence of microvascular antithrombin (AT+) and tPA (tPA+) and absence of arterial endothelial ICAM-1 (ICAM-1). Thrombotic and activated hearts, shown in Figure 1 (bottom row), have myocardial fibrin deposits in capillaries and cardiomyocytes (Fib+), loss of microvascular antithrombin (AT−), loss of arteriolar tPA (tPA−) and expression of arterial endothelial ICAM-1 (ICAM-1+). For predictive models that used data from only the first biopsy, each of these signs was scored either 0, if normal, or 1, if abnormal. For models that used all biopsies obtained during the first three months, we calculated the *proportion* of abnormal signs for each marker (e.g., if a patient had four biopsies and three had fibrin deposits, the marker for fibrin was scored: Fib+ = 3/4 = .75 for that patient). Proportions were re-scaled by a factor of 10 so that regression coefficients could be interpreted as a 10% change in the proportion of abnormal biopsies, a change in approximately one biopsy from normal to abnormal for the typical patient. Four predictors were tested representing the presence or absence of abnormality in the four markers (Fib+, AT−, tPA−, ICAM-1+).

### Statistical model

Univariate logistic regression models were estimated using each biomarker in turn as the sole predictor in the equation. Multivariate stepwise models were then developed in two stages. In stage-one, statistically significant biomarkers were identified by stepwise backward elimination to establish base models. In stage-two, clinical and laboratory covariates shown in Table 1, that were found to be significantly associated with outcome in initial bivariate analyses were forced into the base models. Because time-zero biopsies revealed normal immunohistochemical markers in all cases and exhibited no between-patient variation, they were not considered as potential predictors in any of the regression models.

**Table 1:** Summary of demographic and clinical variables (Patients: n = 172).

VARIABLE	VALUE					
**Donor:**						
Age, mean years (±SD)	28.8					(±11.2)
Sex (percent male)	78.5					
**Recipient:**						
Age, mean years (±SD)	48.7					(±10.2)
Sex (percent male)	66.9					
Race (percent white)	89.5					
Body mass index (kg/m^2^), mean (±SD)	26.5					(±5.0)
Diabetics (%)	40.1					
Insulin dependent diabetics (%)	31.4					
**Reason for transplantation:**						
Coronary artery disease (%)	45.9					
Cardiomyopathy (%)	47.1					
Other (%)	7.0					
**Ischemic time (minutes), mean (±SD)**	156.8					(±56.6)
**Smokers after transplantation (%)**	7.6					
**Hypertensives (%)**	89.0					
**Cholesterol (mmol/l):**						
Total cholesterol, mean (±SD)	5.4					(±1.0)
LDL-C, mean (±SD)	2.6					(±0.8)
HDL-C, mean (±SD)	1.2					(±0.4)
**Number of HLA mismatches:**	0	1	2	3	4	
A/B (%)	0	5.8	16.3	47.1	30.8	
DR (%)	7.00	39.0	54.0			
**Creatinine >123.8 µmol/l (%)**	58.1					
**Ejection fraction, mean (%) (±SD)**	54.3					(±7.4)
**2R-3R rejections (1^st^ 3-mos), mean (±SD)**	0.2					(±0.4)
**Biopsies (1^st^ 3-months), mean (±SD)**	5.2					(±1.0)
**CMV infections (% positive)**	12.8					
**Cell Panel Reactive Antibodies >0% (%)**	8.1					
**Treatment:**						
Prednisone (%)	100.0					
Cyclosporine (%)	94.2					
Azathioprine (%)	68.0					
Mycophenolate mofetil (%)	65.7					
Tacrolimus (%)	11.0					
Sirolimus (%)	7.0					
Statins (%)	77.9					
Calcium Channel Blockers (%)	77.9					
ACE Inhibitors/ARBs (%)	43.0					

All data based on entire sample of 172 patients. Abbreviations: SD: Standard Deviation; LDL-C: low density lipoprotein cholesterol; HDL-C: high density lipoprotein cholesterol; CMV: cytomegalovirus; ACE: Angiotensin-Converting Enzyme; ARB: Angiotensin Receptor Blockers.

### Model cross-validation

Using the method of Austin and Tu [7], regression models were re-estimated for 200 bootstrap [8] samples drawn with replacement from the original data. Bootstrap re-estimation of model parameters is equivalent to performing multiple split-sample validation estimates and provides assessments of model performance without sacrificing sample size [9]. Statistically significant biomarkers identified in stage one and covariates that were found to be univariately associated with outcomes were included in final multivariate models if those variables were retained in ≥60% of the 200 bootstrapped models. A total of 3×3×2 = 18 models were derived to predict one-, five-, and 10-year odds of CAV, severe CAV and graft failure due to CAV using markers from either the first biopsy only, or from all biopsies available at three months.

The Youden Index calculated from receiver operating characteristic (ROC) curves were used to identify optimum cut-off values for the models [10]. Model performance was quantified by evaluating sensitivity, specificity, and predictive accuracy. The C-statistic (area under the ROC curve) was used to quantify discriminative accuracy [11]. Models were further evaluated by comparing the overall percentages of patients correctly classified.

Predicted values from ten-year models were stratified into three groupings: (1) LOW RISK (lower 25% of risk distribution); (2) MODERATE RISK (middle 50%) and (3) HIGH RISK (upper 25%) and separate Kaplan-Meier curves were constructed for these risk groups.

## Results

Demographic and clinical characteristics of the patient population assessed at three months post-transplant are shown in Table 1. Time-zero biopsies performed at the time of transplant but before reperfusion all showed the characteristics of a thromboresistant microvasculature (as illustrated in Figure 1, top row). Because these baseline biopsies were all normal and exhibited no variation they were not considered further in regression models.

When evaluated in univariate regression models (Table 2), individual markers from the first biopsy were statistically significant and possessed good predictive value in most cases (C-statistics: 0.50 to 0.77; odds ratios [ORs]: 0.95 to 13.04). The presence of fibrin emerged as the most common univariate predictor in first-biopsy models. This is in contrast with previous analyses which showed that loss of tPA was the predominant predictor in three-month models [3].

**Table 2:** Univariate logistic regression models using information from the first post-transplant biopsy (N = 172 patients).^a^

		One-Year Risk			Five-Year Risk			Ten-Year Risk		
		(31 cases)^b^			(85 cases)			(106 cases)		
Model	CAV:	C	OR	CI	C	OR	CI	C	OR	CI
1	Fibrin	0.64	3.21	1.46–7.07	0.67	5.39	2.64–11.0	0.67	5.83	2.54–13.4
2	AT	0.64	3.30	1.45–7.50	0.67	4.36	2.29–8.29	0.70	5.68	2.77–11.7
3	tPA	0.63	2.89	0.98–4.79	0.67	4.77	2.44–9.34	0.68	5.60	2.58–12.1
4	ICAM-1	0.59	2.17	1.32–6.36	0.63	3.88	1.90–7.93	0.62	3.76	1.68–8.41

		One-Year Risk			Five-Year Risk			Ten-Year Risk		
		(10 cases)			(36 cases)			(58 cases)		
	Severe CAV:	C	OR	CI	C	OR	CI	C	OR	CI
5	Fibrin	0.75	8.96	1.84–43.7	0.67	4.05	1.87–8.79	0.67	4.38	2.22–8.66
6	AT	0.68	5.26	1.08–25.5	0.66	3.96	1.76–8.91	0.62	2.66	1.39–5.12
7	tPA	0.67	4.07	1.01–16.4	0.65	3.58	1.65–7.75	0.70	5.49	2.76–10.9
8	ICAM-1	0.50	1.02	0.25–4.10	0.62	2.86	1.33–6.17	0.61	2.67	1.35–5.29

		One-Year Risk			Five-Year Risk			Ten-Year Risk		
		(7 cases)			(18 cases)			(31 cases)		
	Failure Due To CAV:	C	OR	CI	C	OR	CI	C	OR	CI
9	Fibrin	0.77	13.04	1.53–111	0.75	8.75	2.73–28.0	0.67	4.14	1.84–9.32
10	AT	0.71	7.75	0.91–65.8	0.71	7.22	2.01–26.0	0.62	2.60	1.16–5.84
11	tPA	0.67	4.26	0.80–22.6	0.69	3.95	1.68–14.6	0.64	3.17	1.42–7.07
12	ICAM-1	0.51	0.95	0.18–5.05	0.64	3.45	1.27–9.33	0.61	2.73	1.23–6.08

Abbreviations: C: C-Statistic (Area under the ROC curve); OR: Odds Ratio; CI: Confidence Intervals; AT: Antithrombin; tPA: tissue Plasminogen Activator; ICAM-1: Intercellular Adhesion Molecule-1.

a Each model uses one biomarker as the single predictor of CAV, severe CAV and failure due to CAV at one-, five-, and ten-years post-transplant.

b Numbers in parentheses (cases) represent the cumulative number of patients experiencing the indicated event at each follow-up interval.

In final multivariate models (Tables 3, 4 and 5), presence of fibrin with or without ICAM-1 expression was the most common statistically significant predictor in first-biopsy models, and loss of tPA was the dominant predictor in three-month models. Once the odds associated with these markers were accounted for, none of the other markers were able to explain additional odds.

**Table 3:** Final multivariate logistic regression models using first-biopsy-only or three-month biopsy data to predict CAV, severe CAV, and graft failure due to CAV at one year post-transplant.

ONE-YEAR RISK of OUTCOME					
CAV:					
Using 1^st^ biopsy, only:			Using all biopsies available at 3 months:		
Predictor Variable	OR	CI	Predictor Variable	OR	CI
Antithrombin	3.30	1.45–7.50	tPA	1.36	1.18–1.57
			Ischemic time	0.99	0.99–1.00

SEVERE CAV:					
Using 1^st^ biopsy, only:			Using all biopsies available at 3 months:		
Predictor Variable	OR	CI	Predictor Variable	OR	CI
Fibrin	30.68	3.91–240.6	tPA	2.22	1.29–3.83
ICAM-1	0.08	0.01–0.60	Ischemic time	0.98	0.96–1.00
Statins	0.05	0.01–0.29	Statins	0.02	0.00–0.17

GRAFT FAILURE DUE TO CAV:					
Using 1^st^ biopsy, only:			Using all biopsies available at 3 months:		
Predictor Variable	OR	CI	Predictor Variable	OR	CI
Fibrin	38.70	4.00–374.8	tPA	1.81	1.08–3.03
ICAM-1	0.13	0.02–0.82			

Abbreviations: OR: Odds Ratio; CI: 95% confidence interval for the OR; tPA: Tissue Plasminogen Activator; ICAM-1: Intercellular Adhesion Molecule-1.

Models on the left use information from the first biopsy only. Models on the right use information from all biopsies available in the first three months post-transplantation.

**Table 4:** Final multivariate logistic regression models using first-biopsy-only or three-month biopsy data to predict CAV, severe CAV, and graft failure due to CAV at five years post-transplant.

FIVE-YEAR RISK of OUTCOME					
CAV:					
Using 1^st^ biopsy, only:			Using all biopsies available at 3 months:		
Predictor Variable	OR	CI	Predictor Variable	OR	CI
Antithrombin	5.34	2.69–10.58	tPA	1.41	1.26–1.58
HLA-AB Mismatch	0.33	0.14–0.75	Recipient Sex (Male)	2.34	1.05–5.19
			HLA-AB Mismatch	0.37	0.15–0.88

SEVERE CAV:					
Using 1^st^ biopsy, only:			Using all biopsies available at 3 months:		
Predictor Variable	OR	CI	Predictor Variable	OR	CI
Fibrin	4.13	1.77–9.68	tPA	1.45	1.23–1.71
MMF Regimen	0.36	0.15–0.84	MMF Regimen	0.45	0.18–1.11
Recipient Sex (Male)	2.22	0.84–5.90	Recipient Sex (Male)	4.17	1.41–12.31
HLA-AB Mismatch	0.41	0.16–1.06	HLA-AB Mismatch	0.33	0.11–0.95
Statins	0.36	0.15–5.90	Statins	0.27	0.10–0.73

GRAFT FAILURE DUE TO CAV:					
Using 1^st^ biopsy, only:			Using all biopsies available at 3 months:		
Predictor Variable	OR	CI	Predictor Variable	OR	CI
Fibrin	9.33	2.34–37.12	tPA	1.73	1.22–2.45
MMF Regimen	0.08	0.02–0.35	MMF Regimen	0.11	0.02–0.49
Statins	0.10	0.03–0.38	Statins	0.06	0.01–0.27

Abbreviations: OR: Odds Ratio; CI: 95% confidence interval for the OR; tPA: Tissue Plasminogen Activator; HLA: Human Leukocyte Antigen; MMF: Mycophenolate Mofetil.

Models on the left use information from the first biopsy only. Models on the right use information from all biopsies available in the first three months post-transplantation.

**Table 5:** Final multivariate logistic regression models using first-biopsy-only or three-month biopsy data to predict CAV, severe CAV, and graft failure due to CAV at ten years post-transplant.

TEN-YEAR RISK of OUTCOME					
CAV:					
Using 1^st^ biopsy, only:			Using all biopsies available at 3 months:		
Predictor Variable	OR	CI	Predictor Variable	OR	CI
Antithrombin	8.73	3.81–20.04	Antithrombin	1.47	1.29–1.68
MMF Regimen	0.37	0.16–0.84	MMF Regimen	0.49	0.21–1.12
Recipient Sex (Male)	2.01	0.93–4.37	Recipient Sex (Male)	2.55	1.12–5.80
HLA-AB Mismatch	0.41	0.17–0.99	HLA-AB Mismatch	0.48	0.19–1.21
Statins	2.12	0.85–5.30	Rejections (1^st^ 3 mo)	0.40	0.13–1.21

SEVERE CAV:					
Using 1^st^ biopsy, only:			Using all biopsies available at 3 months:		
Predictor Variable	OR	CI	Predictor Variable	OR	CI
Fibrin	1.62	0.57–4.59	tPA	1.36	1.21–1.54
tPA	5.86	2.02–17.00	Recipient Sex (Male)	3.53	1.51–8.73
MMF Regimen	0.43	0.20–0.92	HLA-AB Mismatch	0.22	0.09–0.53
HLA-AB Mismatch	2.72	1.14–6.47	Ischemic time	0.99	0.99–1.00
Statins	0.22	0.09–0.54			

GRAFT FAILURE DUE TO CAV:					
Using 1^st^ biopsy, only:			Using all biopsies available at 3 months:		
Predictor Variable	OR	CI	Predictor Variable	OR	CI
Fibrin	3.99	1.53–10.40	tPA	1.31	1.12–1.55
MMF Regimen	0.12	0.04–0.32	MMF Regimen	0.15	0.06–0.41
Statins	0.21	0.08–0.55	Recipient Sex (Male)	2.77	0.88–8.77
			Statins	0.17	0.06–0.47

Abbreviations: OR: Odds Ratio; CI: 95% confidence interval for the OR; tPA: Tissue Plasminogen Activator; HLA: Human Leukocyte Antigen; MMF: Mycophenolate Mofetil.

Models on the left use information from the first biopsy only. Models on the right use information from all biopsies available in the first three months post-transplantation.

The odds ratio (OR) in first-biopsy models represents the multiplicative increase in risk associated with the presence of an abnormal marker. In three-month models the OR is the multiplicative increase in risk associated with a 10% increase in the proportion of abnormal biopsy results (equivalent to approximately one biopsy result over the first three months). As an example, the three-month model predicting graft failure due to CAV at 10 years, which shows an OR for tPA of 1.31 (Table 5), indicates that the 10-year odds of graft failure increases by a factor of 1.31 with each 10% increase in the proportion of biopsies showing loss of tPA in the first three months. A 20% increase in biopsies showing loss of tPA (equivalent to approximately two biopsy results in the first three months) increases the 10-year odds by 1.31×1.31 = 1.7161.

Performance characteristics for all models are summarized in Table 6. Considering positive and negative outcomes together, first-biopsy and three-month models showed similar capacities to classify patients correctly. The one exception was for 10-year predictions of graft failure due to CAV where the three-month model correctly classified a significantly higher percentage of patients (87%) compared to the first-biopsy model (76%).

**Table 6:** Performance characteristics for first-biopsy (First) and three-month (All) biopsy models.^a^

	CAV						SEVERE CAV						GRAFT FAILURE DUE TO CAV					
	At 1 Year		At 5 Years		At 10 Years		At 1 Year		At 5 Years		At 10 Years		At 1 Year		At 5 Years		At 10 Years	
Measure	First	All	First	All	First	All	First	All	First	All	First	All	First	All	First	All	First	All
C (ROC Area)^b^	0.64	0.76	0.72	0.79	0.78	0.81	0.89	0.95	0.77	0.84	0.80	0.78	0.84	0.84	0.92	0.95	0.86	0.88
Sensitivity	0.69	0.81	0.79	0.78	0.62	0.76	0.90	1.00	0.74	0.83	0.74	0.82	0.86	1.00	0.83	0.94	0.87	0.77
Specificity	0.60	0.68	0.58	0.70	0.83	0.75	0.78	0.78	0.72	0.78	0.72	0.70	0.68	0.61	0.85	0.82	0.74	0.89
PPV	0.28	0.37	0.65	0.72	0.86	0.85	0.20	0.22	0.41	0.49	0.57	0.57	0.10	0.10	0.39	0.39	0.43	0.60
NPV	0.89	0.94	0.74	0.76	0.55	0.64	0.99	1.00	0.92	0.95	0.85	0.89	0.99	1.00	0.98	0.99	0.96	0.95
Cutoff Value^c^	0.11	0.21	0.27	0.44	0.68	0.58	0.01	0.03	0.17	0.22	0.31	0.30	0.01	0.02	0.04	0.05	0.12	0.23
Pct Correct^d^	0.62	0.70	0.69	0.74	0.70	0.75	0.79	0.79	0.72	0.79	0.73	0.74	0.69	0.63	0.85	0.83	0.76	0.87^e^
Prevalence^f^	0.19		0.50		0.63		0.06		0.20		0.33		0.04		0.10		0.18	

Abbreviations: PPV: positive predictive value; NPV: negative predictive value.

a First-biopsy (First) and three-month (All) models show similar discriminative and predictive accuracy, particularly with respect to the prediction of severe CAV and graft failure. Negative predictive values (NPV) are particularly high for both first-biopsy and three-month models, indicating that it is possible, using only information from the first biopsy, to identify a patient subgroup at substantially reduced risk of developing long-term CAV or graft failure.

b C: C-statistics: A measure of the area under the receiver operating characteristic (ROC) curve. Equivalently, it is the proportion of all case versus non-case pairs that were correctly classified by the model.

c Cutoff value: The expected value from the logistic regression model that serves as the threshold for predicting the event in question. Patients with expected values that exceed the cutoff are predicted to experience the event.

d Pct Correct is the percent correctly classified by the model and includes both positive and negative classifications.

e Three-month model (All) is significantly better (p<.02) than the first-biopsy model (First) in classifying failure due to CAV at 10 years.

f Prevalence: the proportion of patients that experienced the indicated event by the indicated time point.

Positive predictive values for both first-biopsy and three-month models tended to be low, with the lowest values associated with one-year predictions. By contrast, negative predictive values were high for both sets of models. For example, in first-biopsy models patients with no evidence of fibrin deposition had a 99% chance of avoiding graft failure due to CAV at one year, and continued to have a 98% and 96% chance of being risk-free at five- and 10-years (Table 6). Similarly, patients showing sustained levels of microvascular tPA over the next three-months had a 100% chance of avoiding graft failure due to CAV at one year, and continued to have a 99% and 95% chance of being risk-free at five- and 10-years, respectively. *Thus, the earliest information available from a single biopsy is sufficient to identify a subgroup of patients with very low odds of long-term (10-year) graft failure due to CAV*.

**Figure 2:**
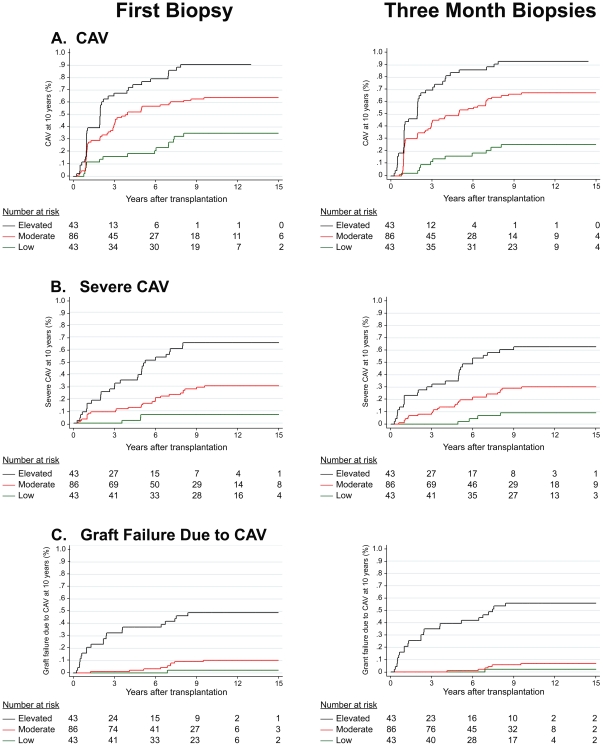
Kaplan-Meier curves using 10-year regression models. Kaplan-Meier curves of risk-stratified groupings derived from first-biopsy versus three-month biopsy models showing time to (a) CAV, (b) severe CAV, and (c) failure due to CAV. Risk groupings were formed from the distributions of the predictive probabilities from 10-year logistic regression models (low risk = lower 25%, moderate risk = middle 50%, and high risk = upper 25%).

Kaplan-Meier curves using risk-stratified predictive values from 10-year regression models (Figure 2) showed significant time-to-event differences by the log-rank test for CAV (p = .001), severe CAV (p = .001), and failure due to CAV (p = .001) for both first-biopsy and three-month models. Thus, first-biopsy models are similar to three-month models not only in their predictions of adverse event incidence, but also in their predictions of *time* to event.

## Discussion

Prediction models using information derived from a single endomyocardial biopsy obtained within a median 9 days post-transplant accurately identified heart transplant patients with *substantially* reduced risk of developing long-term CAV and graft failure.

The high negative predictive accuracies of our models have important clinical implications. First, models that used only the first biopsy had negative predictive values comparable to models that used all biopsies available at three months, confirming our hypothesis that a single early biopsy is sufficient to identify patients with very low risk of long-term (10-year) graft failure due to CAV. This finding implies that it may be possible to reduce the number of follow-up biopsies and coronary angiographies for low-risk patients, provided there is continued absence of symptoms or signs of rejection. Moreover, multiple biopsies during the first three months may be *unnecessary* in patients with no evidence of fibrin deposits in the first biopsy. Of course, patients that are not identified as low-risk would still need to be followed with definitive monitoring. A potential strategy would be to follow low-risk patients non-invasively with gene expression profiling, as has been suggested for the monitoring of patients with low risk for rejection [2], while continuing to follow all other patients using conventional monitoring protocols. In doing so, the complications and costs associated with invasive heart biopsies would be reduced and limited resources could be freed up for more intensive follow-up of higher-risk patients.

The *positive predictive accuracies* of our models were not as strong as the negative predictive accuracies, suggesting that it is harder to identify high-risk than low-risk patients. A likely explanation is that patients with evolving disease are eventually identified and treated preventively during the 10-year follow-up interval, thereby reducing the odds initially predicted based on prior three-month data.

Absence of myocardial fibrin in the first biopsy and persistence of microvascular tPA in all biopsies obtained during the first three months emerged as the best *independent* predictors in most multivariate models, suggesting that the underlying biological process upon which statistical prediction is based evolves over time during the first three months following transplantation. We conclude that early absence of microthrombosis and continued persistence of an intact fibrinolytic system are indicative of a protective phenotype against long-term CAV and allograft failure.

Lack of antithrombin typifies a system that is failing to prevent microvascular fibrin deposits. Thus, it is reasonable that loss of antithrombin in the first-biopsy model would turn out to be the best early predictor of CAV, and that the subsequent unchecked accumulation of microvascular fibrin, secondary to the loss of antithrombin, would be the best early predictor of *severe* CAV and graft failure due to CAV. If microvascular fibrin continues to be deposited due to the failure of antithrombin to prevent it, the patient will still retain some capacity to remove it as long as there is a sustained presence of tPA. However, if there is also loss of tPA, fibrinolytic capacity will be diminished, fibrin deposition will continue unabated, and the patient's status will worsen. Thus, it is reasonable that three-month models would pinpoint loss of tPA as the single best independent predictor of long-term CAV and graft failure.

In first-biopsy models it is noteworthy that ICAM-1 expression, a marker of endothelial activation, appears with fibrin as a co-predictor of severe CAV and graft failure at one year, indicating that endothelial activation in the presence of microvascular fibrin further heightens the odds of very early and very serious CAV. This is consistent with observations from a transient cerebral artery occlusion model showing that concomitant reduction of both ICAM-1 expression and microvascular fibrin significantly reduced brain injury and improved post-ischemic blood flow [12].

The importance of early microthrombosis, reduced fibrinolysis and microvascular arterial endothelial activation for CAV and graft failure has been previously demonstrated [4], [6], [13], [14], [15], [16], [17], [18]. Until now, however, the *sequence* in which these markers emerge during the immediate post-transplant period has not been well described and the prognostic significance of the information contained in the first biopsy has been underappreciated.

The very early appearance of myocardial fibrin suggests that ischemia-reperfusion (I/R) may be a trigger of coagulation activation. The absence of a prothrombotic microvasculature in all time-zero biopsies performed at the time of transplant *and before reperfusion* further confirms that this phenotype developed after the graft was placed into the recipient. These findings rule out the possibility that our risk predictors are a consequence of brain trauma to the donor or damage to the donor heart occurring during the harvesting procedure. Likewise, they increase the likelihood that abnormal markers detected in time-one biopsies are related to I/R occurring immediately after transplantation. I/R appear to damage endothelium by reducing anticoagulation, increasing thrombogenicity and promoting vascular inflammation and microthrombus formation leading to microinfarctions. Massberg et al [19] observed massive ICAM-1-mediated microvascular fibrinogen deposition and platelet adhesion as early as ten minutes after reperfusion in an intestinal I/R model. Furthermore, I/R induce production of reactive oxygen species, promoting endothelial dysfunction and upregulation of ICAM-1 and P-selectin [20]. Interestingly, the fibrin-derived peptide Bβ_15–42_ (FX06) was shown to significantly attenuate I/R injury in a heart transplant model with extended cold ischemia by reducing infiltrating leukocytes [21]. Pathophysiologically, I/R probably promotes endothelial dysfunction and CAV by inducing platelet adhesion, growth factor release, major histocompatibility class I and II antigen upregulation, donor antigen release, and by promoting adhesion molecule expression and smooth muscle cell proliferation [22], [23].

Considering the emergence of antibody-mediated rejection (AMR) as a model of microcirculation injury and endothelial activation [24], [25] and its potential as a predictor of long-term outcome [26], [27], it is relevant to briefly discuss the relationship of I/R with respect to AMR and our own findings [28]. Revelo et al [29] recently showed that the combination of complement components, HLA-DR and fibrin defines AMR in patients at risk for allograft loss from cardiovascular causes and they recognized fibrin as being particularly important for defining severe AMR with a high likelihood of poor patient outcome. I/R may facilitate endothelial susceptibility to a recipient's antibody response leading to further endothelial injury caused by AMR. Since complement and antibody-mediated damage leads to vascular endothelial injury with sometimes puzzling histologic consequences, the additional evaluation of fibrin and HLA-DR over time could help define persistent AMR in the presence of endothelial injury and loss. A hypothesis worth testing is whether patients that develop a prothrombotic microvasculature immediately following transplantation are also more prone to develop AMR. We are at present evaluating C4d and CD68 within the grafts to establish the relationship of those antibody reactivities to the pro- or anti-coagulant status of the microvasculature.

From a prognostic perspective, it is important to ascertain how much predictive information is actually added by incorporating a new marker into the best current model, since new markers may contain little or no additional information not already conveyed by existing factors optimally combined [30]. For this reason, the predictive accuracy of AMR-related factors should be judged by comparing the best current model, with and without the AMR-related factors included, using an overall indicator of model discrimination such as the area under the ROC curve as the criteria for judging the degree of improvement [31], [32]. Since we have shown here that our models have excellent *negative* predictive accuracy, it would be especially important to know whether the *positive* predictive accuracy of our models could be significantly improved by adding AMR-related factors.

Our data suggest that graft failure may depend upon the extent of very early post-transplant microvascular damage and the capacity of the transplanted heart to remove microthrombi through active fibrinolysis. Thus, therapies designed to de-escalate hypercoagulability may be most effective if applied during the pre- to peri- and early post-operative periods.

Our study has strengths and weaknesses. Weaknesses include the utilization of angiography rather than intravascular ultrasound [33] and the lack of baseline angiograms at the time of transplantation. From a statistical point of view, our prediction models ultimately need to be tested in other populations by other investigators working in other settings in order to evaluate their generalizability. However, our models did undergo cross-validation on repeated bootstrap samples. Cross-validation produces estimates of a model's likely performance on future data and greatly reduces the likelihood of spurious variable selection that is often the most important source of bias arising from stepwise regression on a single sample [9]. Strengths include the relatively large number of transplant patients, the long multi-year follow-up, and the availability of a large immunohistochemical heart-biopsy database.

The most important clinical message that emerges from our data is that first-biopsy models are comparable to three-month models as evidenced by their similar capacities to classify patients correctly and to single out patients at low risk of CAV and graft failure. The only exception was the superior performance of the three-month model to correctly classify patients with respect to 10-year graft failure due to CAV. The high negative predictive accuracies associated with both, first-biopsy and three-month models are especially noteworthy. Absence of a prothrombotic microvasculature, even when observed as early as 9 days post-transplant in a single biopsy, identifies patients that rarely develop CAV and graft failure. These very low-risk patients are unlikely to derive benefit from further invasive monitoring. Since repeated heart biopsy procedures are both risky and expensive, our findings have implications for both patients and payers. Of course, patients that do not fall within this low-risk group should continue to be followed under standard protocols using more definitive (invasive) monitoring.

Although our findings show that it is possible, using markers that are available within days of the transplant procedure, to identify a subgroup of patients that almost never develops long-term CAV or graft failure, they do not show what the impact on patient outcomes would be if physicians used our prediction models as a clinical tool to manage their transplant patients. Clinical impact can only be demonstrated in a prospective outcome trial in which some patients are randomly assigned to receive usual care and others to a protocol that uses our models to guide treatment decisions.
